# HIV1 drug resistance among patients experiencing first-line treatment failure in Ethiopia: protocol for a systematic review and meta-analysis

**DOI:** 10.1186/s13643-024-02605-1

**Published:** 2024-07-15

**Authors:** Melashu Balew, Gedefaw Abeje, Alemtsehay Mekonnen, Getu Degu

**Affiliations:** 1 School of Public Health, College of Medicine and Health Sciences, Bahir Dar University, Bahir Dar, Ethiopia; 2 Health Research Development Directorate, Amhara Public Health Institute, Bahir Dar, Ethiopia

**Keywords:** HIV, Drug resistance, Treatment failure, Ethiopia

## Abstract

**Background:**

The emergence of HIV drug resistance presents a substantial challenge. Current antiretroviral treatments, along with current classes, face the danger of becoming partially or entirely inactive. As a result, alternative treatment regimens are limited, and treatment choices are complicated. According to the recommendation of the WHO, nations should consider changing their first-line ART regimen if HIV drug resistance exceeds 10%. In spite of the fact that a number of primary studies have been performed on HIV drug resistance in Ethiopia, their pooled prevalence rate has not been determined in a systematic review and meta-analysis, which may provide stronger evidence. Therefore, the objective of this systematic review and meta-analysis will be to estimate the pooled prevalence rate of HIV1 drug resistance in patients with first-line treatment failure in Ethiopia.

**Methods:**

Primary studies will be identified from PubMed/MEDLINE, Scopus, Embase, Web of Science Core Collection, and Google Scholar. The period of search will be from 01 April to 30 June 2024. Studies identified through the search strategies will first be screened by titles and abstracts. Included studies meeting established criteria will be evaluated for risk of bias using the JBI checklist. Data will be extracted, and the pooled prevalence rate of HIV drug resistance will be computed using STATA 14 software. Random effect models will be used when heterogeneity is suspected. The *I*^2^ statistic and its corresponding *P* value will be checked to distinguish heterogeneity. Additionally, publication bias and heterogeneity will be checked using visual funnel plots, Egger’s test, trim-and-fill tests, meta-regression, and subgroup analysis. To present and synthesize the results, narrative synthesis will be performed to describe study characteristics and findings, and forest plots will be used to visually represent effect sizes and confidence intervals from individual studies.

**Discussion:**

Estimating the pooled prevalence rate of HIV drug resistance through a systematic review and meta-analysis improves the reliability of the evidence, the availability of effective HIV treatment options, and the ability to assist in making decisions for both clinical practice and public health policy in Ethiopia.

**Systematic review registration:**

PROSPERO CRD42024533975.

**Supplementary Information:**

The online version contains supplementary material available at 10.1186/s13643-024-02605-1.

## Background

The increase in HIV drug resistance is a major challenge to HIV/AIDS treatment. The main cause is the rapid mutation of the virus, which has a significant impact on the efficacy of antiretroviral therapy [1]. Various types of resistance, including pretreatment, acquired resistance, and cross-resistance, can develop due to factors such as poor treatment compliance, inadequate drug concentrations, or the spread of variant strains drug resistance [2–4].

Patients with treatment failure (two consecutive viral load results > 1000 copies/mL) are at higher risk of HIV drug resistance (a consequence of mutations occurring in viral proteins that antiretroviral drugs target) [5, 6]. Current antiretroviral treatments, as well as newer drugs, face the risk of becoming partially or completely ineffective in the presence of drug-resistant strains of the virus [7].

Due to their cost-effectiveness, non-nucleoside reverse transcriptase inhibitors (NNRTIs), such as efavirenz and nevirapine, as well as nucleoside reverse transcriptase inhibitors (NRTIs), such as tenofovir and lamivudine, are utilized as first-line treatments in many resource-limited settings [8]. However, the emergence of resistance to NNRTIs and/or NRTIs poses a significant challenge as it reduces available alternatives for effective treatment regimens and further complicates treatment strategies [1, 9]. According to WHO recommendation, countries should consider changing first-line ART regimen if HIV drug resistance rates exceed 10% [10].

HIV drug resistance has three levels: low, medium, and high. High levels of drug resistance highlight the urgent need for alternative treatment options. Therefore, concerns about the spread of drug-resistant strains are increasing. On the other hand, the presence of low and moderate levels of resistance emphasizes the need for early diagnosis and prevention of resistance, thereby ensuring the effectiveness of current antiretroviral drugs [4, 11–13]. There are many risk factors for HIV drug resistance. Irregular and repeated interruption, transmission of drug-resistant strains, delay in reporting treatment failure, use of incomplete or inadequate treatment regimens, and exposure to antiretroviral drugs previously increased the risk of HIV drug resistance emerging [14–16].

Africa, which has the world’s largest HIV/AIDS burden [17], also faces challenges related to drug resistance. Recent studies conducted many African countries have consistently highlighted the impact of HIV drug resistance in patients with treatment failure. A study conducted in HIV-infected adolescents in the two main cities of Cameroon found that 93.3% HIVDR, of which 52.3% were high-level resistance to efavirenz/nevirapine (K103N) and 47.6% to lamivudine (M184V) [18]; 52.7% of participants in Kenya had at least one resistance mutation [19], and 83% of the patients in Zambia required a drug change in adolescents and young adults who had at least one drug resistance mutation [20].

In Ethiopia, where HIV is prevalent, free antiretroviral therapy (ART) has been provided since 2005 [21]. In 2022, 610000 [510000-750000] people were living with HIV, and 504685 patients received ART [22]. According to national guidelines, TDF + 3TC + DTG (FDC) is the preferred first-line regimen and TDF/AZT + 3TC + EFV or AZT + 3TC + DTG is the alternative first-line regimen for patients aged 10 years and older. Moreover, ABC + 3TC + DTG is the preferred first-line regimen, and ABC + 3TC + LPV/r or AZT + 3TC + DTG is the alternative first-line regimen for children under 10 years of age [23].

However, different studies have shown that of HIV resistance rates vary. The presence of two studies with different findings (5% and 85.4%) [24, 25] indicated a significant challenge for HIV management and requires careful evaluation of alternate treatment options for viral suppression. Although these studies may provide valuable information, a systematic review and meta-analysis of their pooled prevalence has not yet been performed, which could provide stronger evidence to draw conclusions for broader populations [26]. As a result, there are gaps in understanding the extent of drug resistance in patients who experience first-line treatment failure.

Addressing this gap is critical to inform evidence-based policies and strategies to improve HIV treatment and care in Ethiopia. Systematic reviews and meta-analysis, which synthesize data from a variety of sources and apply rigorous analytical methods, can improve the strength and reliability of the evidence, thereby providing valuable insights for clinical practice and public health policy development in Ethiopia. Therefore, the objective of this systematic review and meta-analysis will be to estimate the pooled prevalence rate of HIV drug resistance among patients experiencing treatment failure in Ethiopia.

## Methods

This protocol was registered on PROSPERO on April 21, 2024, with the identification number CRD42024533975. As shown in Additional file 1, the protocol was written according to the Preferred Reporting Items for Systematic Reviews and Meta-Analyses Protocols (PRISMA-P) guidelines [27]. The systematic review and meta-analyses will be performed based on the JBI Manual for Evidence Synthesis [28].

### Eligibility criteria

Studies conducted in Ethiopia, including published primary studies with cross-sectional or cohort designs reporting HIV drug resistance, will be included. However, studies that do not present clear data, qualitative studies, editorials, or commentaries will be excluded because the inability to report prevalence data.

The CoCoPop (Condition, Context, and Population) mnemonic will be used to include relevant studies. In this study, the condition will be HIV drug resistance, the context will be Ethiopia, and the population will be patients who have experienced first-line treatment failure.

### Information sources

We will search electronically from PubMed/MEDLINE, Scopus, Embase, Web of Science Core Collection, Google Scholar, and manually from Google to identify published literature. The period of search will be from April 1 to June 30, 2024. A comprehensive search strategy will be developed. Free text (keywords), entry terms, medical subject headings (MeSH) with respect to CoCoPop mnemonics, a combination of keywords related to HIV, drug resistance, Ethiopia, Africa South of the Sahara, and developing countries will be used. Using Boolean operators, search terms will be generated to find relevant studies from PubMed, Scopus, and Google Scholar. Search queries are provided in Additional file 2. Moreover, the reference lists of the included papers will be screened to identify relevant articles.

### Data management

Endnote version 20.4 will be used to manage the selection and review process. Articles from the database search will be imported into Endnote. Duplicate articles will be removed and relevant studies will be included to synthesize quantitative data. Data will be extracted using Microsoft Excel, and the meta-analyses will be performed in STATA version 14.

### Selection process

Screening will first be performed independently by two reviewers using titles and abstracts. Full-text articles meeting the inclusion criteria will be read independently by the reviewers for further assessment. Prioritization method will be used to exclude articles the full text screening phase based on the following criteria: relevance to the review question, geographic region, study design, article type, and comorbidity of participants [29]. Disagreements will be resolved by consensus between the two reviewers and a third reviewer if consensus has not been reached to include or reject the article in the systematic review and meta-analysis.

### Data collection process and data items

Two reviewers will independently extract data from selected full-text articles using a designed Microsoft Excel extraction format. Then, the reviewers will compare the data to ensure consistency. Inconsistencies will be resolved by discussion between the two data extractors, and disagreements will be resolved by involving a third reviewer.

Data relevant to this study will be collected from each study. The following data items will be extracted: name of author, publication year, region, population type, study design, study duration, number of participants, demographics (age and sex), results by outcomes of interest (outcome definition, sample size, and statistical tests), NRTI-associated mutations, NNRTI-associated mutations, HIV-1 subtype distribution, and regimen type (Additional file 3).

### Outcomes and prioritization

The outcome variable of this systematic review and meta-analysis will be the pooled prevalence rate of HIV drug resistance, calculated as the total number of patients who develop HIV drug resistance divided by the total number of patients with first-line treatment failure in each included study and multiplied by 100.

### Study risk of *bias* assessment

To ensure methodological quality, two reviewers will independently assess the quality of the included studies. We will assess risk of bias using the Joanna Briggs Institute (JBI) critical appraisal checklist, a comprehensive tool designed to assess quality and risk of bias in various types of study designs. Each checklist includes a series of questions such as the clarity of the research question, the rigor of data collection and analysis, and the validity and reliability of the results, thereby helping improve the reliability and validity of synthesized evidence. The various biases will be evaluated using the JBI critical appraisal checklist. The questions in the checklist address selection bias by examining how participants are recruited, performance bias by examining blinding methods, and detection bias by assessing the outcomes assessed. Questions related to attrition bias focus on handling incomplete data, while reporting bias is assessed by examining whether all predetermined outcomes are reported consistently. This systematic approach ensures thorough assessment of potential biases contributing to the overall robustness of the meta-analysis [28, 30]. Two reviewers will independently assess the quality of included studies using the checklist. During the evaluation, disagreements between reviewers will be resolved through discussion or through the participation of other reviewers.

### Effect measures and synthesis methods

A narrative synthesis that includes a summary of main characteristics, results of the included studies and discussing potential reasons for differences in study outcomes will be performed to summarize review findings. Additionally, meta-analyses will be performed in STATA (V.14) using the “metan” command. DerSimonian and Laird’s (DL) overall pooled prevalence rate of HIV drug resistance in patients experiencing first-line treatment failure will be calculated as an effect measure.

This meta-analysis will be performed considering studies with low risk of bias and heterogeneity. An overall estimate of HIV drug resistance will be calculated and summarized in a forest plot. Random effects models will be used if heterogeneity is suspected. Heterogeneity will be tested using the *I*^2^ statistic, which measures the proportion of variance due to inconsistency. An *I*^2^ value of 0% indicates no inconsistency between the results of individual studies. Low, medium, and high heterogeneity will be considered with *I*^2^ values of 25%, 50%, and 75%, respectively [31].

*I*^2^, subgroup analysis, and meta-regression will be performed to investigate the source of statistical heterogeneity. A sensitivity analysis will also be performed on HIV drug resistance rates to assess the impact of attrition bias.

### *Meta*-*bias*(es)

Publication bias (small study bias or assessment of reporting bias) will be checked by visual inspection of the funnel plot (the effect size against its standard error) and statistically using Egger’s test (*P* < 0.05 to declare potential publication bias) and the trim-and-fill method [32].

### Confidence in cumulative evidence

In this systematic review and meta-analysis, the strength of evidence will be assessed using the GRADE (Grading of Recommendations, Assessment, Development, and Evaluation) approach. The overall quality of the evidence in the studies will be classified into four levels: high, moderate, low, and very low. It will take into account a number of factors, including risk of bias, inconsistent results, indirect evidence, imprecise results, and the suggestion of spurious effects when results show no effect [33, 34]. Applying the GRADE approach, we will systematically evaluate each of these factors for each outcome in the studies included in the review.

## Discussion

HIV’s rapid replication, combined with error-prone reverse transcriptase, leads to significant genetic diversity, creating many viral variants within a single host. This genetic diversity is further complicated by the presence of many different strains, including major groups, subtypes, and circulating recombinant forms. Together, these factors complicate treatment and prevention efforts by increasing the risk of drug resistance. The aim of this review is to generate consolidated evidences on the emergence of HIV1 drug resistance in Ethiopia, which is essential to suppress the virus and maintain effective control measures.

Additionally, NRTI-associated mutations, NNRTI-associated mutations, and HIV-1 subtype distribution will be described. Information on these issues is very important for managing patients with treatment failure and requires careful evaluation of alternate treatment options for viral suppression.

Overall, estimating the pooled prevalence rate of HIV drug resistance through systematic review and meta-analysis improves the reliability of the evidence, the availability of effective HIV treatment options, and ability to assist in decision-making in clinical practice and public health policy in Ethiopia.

### Supplementary Information

Supplementary Material 1.

Supplementary  Material 2.

Supplementary Material 3.

## Data Availability

Not applicable.

## Abbreviations

AIDS: Acquired immunodeficiency syndrome
CoCoPop: Condition, Context, and Population
GRADE: Grading of Recommendations, Assessment, Development, and Evaluation
HIV: Human immunodeficiency virus
HIVDR: Human immunodeficiency virus drug resistance
JBI: Joanna Briggs Institute
MeSH: Medical subject headings
NNRTIs: Non-nucleoside reverse transcriptase inhibitors
NRTIs: Nucleoside reverse transcriptase inhibitors
PRISMA-P: Preferred Reporting Items for Systematic Review and Meta-Analysis Protocols
PROSPERO: The International Prospective Register of Systematic Reviews
