# Supplementary material for: HIV1 drug resistance among patients experiencing first-line treatment failure in Ethiopia: protocol for a systematic review and meta-analysis
Source: Syst Rev. 2024 Jul 15;13:180. doi: 10.1186/s13643-024-02605-1 (PMC11247858; doi:10.1186/s13643-024-02605-1)
Supplement: Supplementary file 2 — Supplementary Material 2. [file 13643_2024_2605_MOESM2_ESM.docx]

# **Additional file 2_Logic grid and search strategy**

# **Logic grid**

This logic grid follows the CoCoPop (Condition, Context, Population) framework. The grid utilizes keywords from our title. The condition (HIV drug resistance), context (Ethiopia) and population (patients experiencing first-line treatment failure) will be effectively applied for searching of literature from databases (Table 1).

Table 1: Logic grid (CoCoPoP)

| Condition | | Context | Population |
| --- | --- | --- | --- |
| HIV | drug resistance |  |  |
| - Human Immunodeficiency Virus - Immunodeficiency Virus$, Human - Virus$, Human Immunodeficiency - Human Immunodeficiency Viruses - Human T Cell Lymphotropic Virus Type III - Human T-Cell Lymphotropic Virus Type III - Human T-Cell Leukemia Virus Type III - Human T Cell Leukemia Virus Type III - LAV-HTLV-III - Lymphadenopathy-Associated Virus - Lymphadenopathy Associated Virus$ - Virus$, Lymphadenopathy-Associated - Human T Lymphotropic Virus Type III - Human T-Lymphotropic Virus Type III - AIDS Virus$ - Virus$, AIDS - Acquired Immune Deficiency Syndrome Virus - Acquired Immunodeficiency Syndrome Virus - HTLV-III - HIV [MeSH] | - Drug resistance - Resistance, Drug - **Drug Resistance (MeSH)** - antiretroviral resistance - antiretroviral therapy resistance - Highly Active Antiretroviral Therapy resistance - HAART resistance | - **Ethiopia (MeSH)** - Federal Democratic Republic of Ethiopia - Addis Ababa - Bahir Dar - Hawassa - Gondar - Amhara - Mekele - Tigrai - Oromia - SNNPR - Afar - Somali - Sub-Saharan Africa - Subsaharan Africa - Africa, Sub-Saharan - **Africa South of the Sahara (MeSH)** - Developing Country - Developing Nation$ - Least Developed Countr$ - Country, Least Developed - Developed Country, Least - Country, Less-Developed - Less Developed Countries - Less-Developed Countr$ - LMICs - Countries, Third-World - Country, Third-World - Third World Countries - Third-World Countr$ - Nation, Third-World - Third World Nations - Third-World Nation$ - Country, Under-Developed - Under Developed Countries - Under-Developed Countr$ - Nation, Under-Developed - Under Developed Nations - Under-Developed Nation$ - Less-Developed Nation$ - Less Developed Nations - Nation, Less-Developed - Country, Low Income - Low Income Countr$ - **Developing Countries (MeSH)** | Patients experiencing first line treatment failure |

# **Search strategy**

Our search strategy will be based on the following keywords: HIV drug resistance, Ethiopia. A query is created for each key word and overall search phrase utilizing Boolean operators (AND, OR, and NOT) to retrieve the required articles from databases (PubMed, SCOPUS, and Google Scholar). In addition, we will record the time and date of the search (Table 2-4).

Table 2: Search strategy from PubMed/Medline

| Search | Query |
| --- | --- |
| HIV | (((((((((((((((((((((((((Human Immunodeficiency Virus) OR (Immunodeficiency Virus, Human)) OR (Immunodeficiency Viruses, Human)) OR (Virus, Human Immunodeficiency)) OR (Viruses, Human Immunodeficiency)) OR (Human Immunodeficiency Viruses)) OR (Human T Cell Lymphotropic Virus Type III)) OR (Human T-Cell Lymphotropic Virus Type III)) OR (Human T-Cell Leukemia Virus Type III)) OR (Human T Cell Leukemia Virus Type III)) OR (LAV-HTLV-III)) OR (Lymphadenopathy-Associated Virus)) OR (Lymphadenopathy Associated Virus)) OR (Lymphadenopathy Associated Viruses)) OR (Virus, Lymphadenopathy-Associated)) OR (Viruses, Lymphadenopathy-Associated)) OR (Human T Lymphotropic Virus Type III)) OR (Human T-Lymphotropic Virus Type III)) OR (AIDS Virus)) OR (AIDS Viruses)) OR (Virus, AIDS)) OR (Viruses, AIDS)) OR (Acquired Immune Deficiency Syndrome Virus)) OR (Acquired Immunodeficiency Syndrome Virus)) OR (HTLV-III)) OR (HIV[MeSH Terms]) |
| Resistance | ((((((Drug resistance) OR (Resistance, Drug)) OR (Drug Resistance [MeSH Terms])) OR (antiretroviral resistance)) OR (antiretroviral therapy resistance)) OR (Highly Active Antiretroviral Therapy resistance)) OR (HAART resistance) |
| Ethiopia | (((((((((((((((((((((((((((((((((((((((((((((((((((((((((((((Ethiopia) OR (Ethiopia[MeSH Terms])) OR (Federal Democratic Republic of Ethiopia)) OR (Addis Ababa)) OR (Bahir Dar)) OR (Bahirdar)) OR (Addis Abeba)) OR (Hawassa)) OR (Awassa)) OR (Gondar)) OR (Gonder)) OR (Amhara)) OR (Mekele)) OR (Tigrai)) OR (Tigray)) OR (Oromia)) OR (SNNPR)) OR (Southern Nation Nationality People Region)) OR (Afar)) OR (Somali region)) OR (Ethiopia Somali Region)) OR (Sub-Saharan Africa)) OR (Subsaharan Africa)) OR (Africa, Sub-Saharan)) OR (Africa South of the Sahara[MeSH Terms])) OR (Developing Country)) OR (Developing Nation)) OR (Developing Nations)) OR (Least Developed Country)) OR (Least Developed Countries)) OR (Country, Least Developed)) OR (Developed Country, Least)) OR (Country, Less-Developed)) OR (Less Developed Countries)) OR (Less-Developed Country)) OR (Less-Developed Countries)) OR (LMICs)) OR (Countries, Third-World)) OR (Country, Third-World)) OR (Third World Countries)) OR (Third-World Country)) OR (Third-World Countries)) OR (Nation, Third-World)) OR (Third World Nations)) OR (Third-World Nations)) OR (Third-World Nation)) OR (Country, Under-Developed)) OR (Under Developed Countries)) OR (Under-Developed Country)) OR (Under-Developed Countries)) OR (Nation, Under-Developed)) OR (Under Developed Nations)) OR (Under-Developed Nations)) OR (Under-Developed Nation)) OR (Less-Developed Nations)) OR (Less-Developed Nation)) OR (Less Developed Nations)) OR (Nation, Less-Developed)) OR (Country, Low Income)) OR (Low Income Country)) OR (Low Income Countries)) OR (Developing Countries[MeSH Terms]) |
| Over all search | (((((((((((((((((((((((((((Human Immunodeficiency Virus) OR (Immunodeficiency Virus, Human)) OR (Immunodeficiency Viruses, Human)) OR (Virus, Human Immunodeficiency)) OR (Viruses, Human Immunodeficiency)) OR (Human Immunodeficiency Viruses)) OR (Human T Cell Lymphotropic Virus Type III)) OR (Human T-Cell Lymphotropic Virus Type III)) OR (Human T-Cell Leukemia Virus Type III)) OR (Human T Cell Leukemia Virus Type III)) OR (LAV-HTLV-III)) OR (Lymphadenopathy-Associated Virus)) OR (Lymphadenopathy Associated Virus)) OR (Lymphadenopathy Associated Viruses)) OR (Virus, Lymphadenopathy-Associated)) OR (Viruses, Lymphadenopathy-Associated)) OR (Human T Lymphotropic Virus Type III)) OR (Human T-Lymphotropic Virus Type III)) OR (AIDS Virus)) OR (AIDS Viruses)) OR (Virus, AIDS)) OR (Viruses, AIDS)) OR (Acquired Immune Deficiency Syndrome Virus)) OR (Acquired Immunodeficiency Syndrome Virus)) OR (HTLV-III)) OR (HIV[MeSH Terms])) AND (((((((Drug resistance) OR (Resistance, Drug)) OR (Drug Resistance [MeSH Terms])) OR (antiretroviral resistance)) OR (antiretroviral therapy resistance)) OR (Highly Active Antiretroviral Therapy resistance)) OR (HAART resistance))) AND ((((((((((((((((((((((((((((((((((((((((((((((((((((((((((((((Ethiopia) OR (Ethiopia[MeSH Terms])) OR (Federal Democratic Republic of Ethiopia)) OR (Addis Ababa)) OR (Bahir Dar)) OR (Bahirdar)) OR (Addis Abeba)) OR (Hawassa)) OR (Awassa)) OR (Gondar)) OR (Gonder)) OR (Amhara)) OR (Mekele)) OR (Tigrai)) OR (Tigray)) OR (Oromia)) OR (SNNPR)) OR (Southern Nation Nationality People Region)) OR (Afar)) OR (Somali region)) OR (Ethiopia Somali Region)) OR (Sub-Saharan Africa)) OR (Subsaharan Africa)) OR (Africa, Sub-Saharan)) OR (Africa South of the Sahara[MeSH Terms])) OR (Developing Country)) OR (Developing Nation)) OR (Developing Nations)) OR (Least Developed Country)) OR (Least Developed Countries)) OR (Country, Least Developed)) OR (Developed Country, Least)) OR (Country, Less-Developed)) OR (Less Developed Countries)) OR (Less-Developed Country)) OR (Less-Developed Countries)) OR (LMICs)) OR (Countries, Third-World)) OR (Country, Third-World)) OR (Third World Countries)) OR (Third-World Country)) OR (Third-World Countries)) OR (Nation, Third-World)) OR (Third World Nations)) OR (Third-World Nations)) OR (Third-World Nation)) OR (Country, Under-Developed)) OR (Under Developed Countries)) OR (Under-Developed Country)) OR (Under-Developed Countries)) OR (Nation, Under-Developed)) OR (Under Developed Nations)) OR (Under-Developed Nations)) OR (Under-Developed Nation)) OR (Less-Developed Nations)) OR (Less-Developed Nation)) OR (Less Developed Nations)) OR (Nation, Less-Developed)) OR (Country, Low Income)) OR (Low Income Country)) OR (Low Income Countries)) OR (Developing Countries[MeSH Terms])) |

Table 3: Search strategy from SCOPUS

| Search | Query |
| --- | --- |
| HIV | TITLE-ABS-KEY ( hiv OR "Human Immunodeficiency Virus*" OR "Acquired Immunodeficiency Syndrome Virus" ) |
| Drug resistance | TITLE-ABS-KEY ( "HIV drug resistance" OR "Resistance, Drug" OR "antiretroviral resistance" OR "antiretroviral therapy resistance" OR "Highly Active Antiretroviral Therapy resistance" OR "HAART resistance" ) |
| Ethiopia | TITLE-ABS-KEY ( ethiopia OR "Federal Democratic Republic of Ethiopia" OR "Addis Ababa" OR "Bahir Dar" OR bahirdar OR "Addis Abeba" OR hawassa OR gondar OR gonder OR amhara OR mekele OR tigrai OR tigray OR oromia OR snnpr OR "Southern Nation Nationality People Region" OR afar OR "Somali region" OR "Ethiopia Somali Region" OR "Sub-Saharan Africa" OR "low income countr*" OR "East Africa" ) |
| Over all search | TITLE-ABS-KEY ( hiv OR "Human Immunodeficiency Virus*" OR "Acquired Immunodeficiency Syndrome Virus" ) AND TITLE-ABS-KEY ( "HIV drug resistance" OR "Resistance, Drug" OR "antiretroviral resistance" OR "antiretroviral therapy resistance" OR "Highly Active Antiretroviral Therapy resistance" OR "HAART resistance" ) AND TITLE-ABS-KEY ( ethiopia OR "Addis Ababa" OR "Addis Abeba" OR hawassa OR gondar OR gonder OR amhara OR oromia OR "Southern Nation Nationality People Region" OR afar OR "Somali region" OR "Ethiopia Somali Region" OR "Sub-Saharan Africa" OR "low income countr*" OR "East Africa" ) AND ( LIMIT-TO ( DOCTYPE , "ar" ) OR LIMIT-TO ( DOCTYPE , "cp" ) ) |
