# Supplementary material for: HIV1 drug resistance among patients experiencing first-line treatment failure in Ethiopia: protocol for a systematic review and meta-analysis
Source: Syst Rev. 2024 Jul 15;13:180. doi: 10.1186/s13643-024-02605-1 (PMC11247858; doi:10.1186/s13643-024-02605-1)
Supplement: Supplementary file 3 — Supplementary Material 3. [file 13643_2024_2605_MOESM3_ESM.docx]

# **Data extraction instruments**

Data extraction comprises details regarding selected full-text papers (study design, sample size, and year of publication), research population characteristics (sex, age range, population type), and the main outcome variable (prevalence rate of HIV drug resistance). Furthermore, the distributions of NRTI and NNRTI-associated mutations, as well as HIV-1 subtypes, will be retrieved using the formats presented below (Table 6-8).

Table 6: Characteristics of selected studies, 2024

| Articles | Year of publication | Prevalence rate of drug resistance | Study design | Sample size | Population | Region | Number of females | Age range |
| --- | --- | --- | --- | --- | --- | --- | --- | --- |
|  |  |  |  |  |  |  |  |  |
|  |  |  |  |  |  |  |  |  |
|  |  |  |  |  |  |  |  |  |

Table 7: NRTI-associated mutations

| **Articles** | M184 IV | K65R | A62V | K70R/E | Y115F | T215Y/F/I | D67G/N | K219Q/E/N | L74V/I | M41L |
| --- | --- | --- | --- | --- | --- | --- | --- | --- | --- | --- |
|  |  |  |  |  |  |  |  |  |  |  |
|  |  |  |  |  |  |  |  |  |  |  |
|  |  |  |  |  |  |  |  |  |  |  |

Table 8: NNRTI-associated mutations

| **Articles** | K103N/S | Y181C | V106A/M | V108I | Y188C | G190A/E/S | K101E/P | P225H | M230L | Y181S | Y188L | V90I |
| --- | --- | --- | --- | --- | --- | --- | --- | --- | --- | --- | --- | --- |
|  |  |  |  |  |  |  |  |  |  |  |  |  |
|  |  |  |  |  |  |  |  |  |  |  |  |  |
|  |  |  |  |  |  |  |  |  |  |  |  |  |
|  |  |  |  |  |  |  |  |  |  |  |  |  |

Table 9: HIV-1 subtype distribution

| **Articles** | subtype C | Subtypes A1 | Subtypes D | Recombinant D_A1 | Recombinant C_A1 | CRF-07_BC | CRF-02_AG | M-09-CPX | BF1 |
| --- | --- | --- | --- | --- | --- | --- | --- | --- | --- |
|  |  |  |  |  |  |  |  |  |  |
|  |  |  |  |  |  |  |  |  |  |
|  |  |  |  |  |  |  |  |  |  |
